# Laboratory Information Management Software for genotyping workflows: applications in high throughput crop genotyping

**DOI:** 10.1186/1471-2105-7-383

**Published:** 2006-08-17

**Authors:** B Jayashree, Praveen T Reddy, Y Leeladevi, Jonathan H Crouch, V Mahalakshmi, Hutokshi K Buhariwalla, KE Eshwar, Emma Mace, Rolf Folksterma, S Senthilvel, Rajeev K Varshney, K Seetha, R Rajalakshmi, VP Prasanth, Subhash Chandra, L Swarupa, P SriKalyani, David A Hoisington

**Affiliations:** 1 Bioinformatics and Biometrics Unit, International Crops Research Institute for the Semi-Arid Tropics (ICRISAT), Patancheru 502 324, Andhra Pradesh, India; 2 Applied Genomics Laboratory, ICRISAT, Patancheru 502 324, Andhra Pradesh, India; 3 International Wheat and Maize Improvement Centre (CIMMYT), Apdo. Postal 6-641, 06600 México, D.F., México; 4 3-6-502, Satguru Apts, Himayat Nagar, Hyderabad 500029, Andhra Pradesh, India; 5 c/o International Wheat and Maize Improvement Centre (CIMMYT), Apdo. Postal 6-641, 06600 México, D.F., México; 6 Hermitage Research Station, Queensland Department of Primary Industries and Fisheries, Yangan Rd, via Warwick Q 4370 Australia; 7 De Ruiter Seeds Benelux, P.O. Box 1050, 2660 BB Bergschenhoek, The Netherlands

## Abstract

**Background:**

With the advances in DNA sequencer-based technologies, it has become possible to automate several steps of the genotyping process leading to increased throughput. To efficiently handle the large amounts of genotypic data generated and help with quality control, there is a strong need for a software system that can help with the tracking of samples and capture and management of data at different steps of the process. Such systems, while serving to manage the workflow precisely, also encourage good laboratory practice by standardizing protocols, recording and annotating data from every step of the workflow.

**Results:**

A laboratory information management system (LIMS) has been designed and implemented at the International Crops Research Institute for the Semi-Arid Tropics (ICRISAT) that meets the requirements of a moderately high throughput molecular genotyping facility. The application is designed as modules and is simple to learn and use. The application leads the user through each step of the process from starting an experiment to the storing of output data from the genotype detection step with auto-binning of alleles; thus ensuring that every DNA sample is handled in an identical manner and all the necessary data are captured. The application keeps track of DNA samples and generated data. Data entry into the system is through the use of forms for file uploads. The LIMS provides functions to trace back to the electrophoresis gel files or sample source for any genotypic data and for repeating experiments. The LIMS is being presently used for the capture of high throughput SSR (simple-sequence repeat) genotyping data from the legume (chickpea, groundnut and pigeonpea) and cereal (sorghum and millets) crops of importance in the semi-arid tropics.

**Conclusion:**

A laboratory information management system is available that has been found useful in the management of microsatellite genotype data in a moderately high throughput genotyping laboratory. The application with source code is freely available for academic users and can be downloaded from .

## Background

Plant genotyping is a technology that has widespread applications in the fields of breeding, research and commerce. With the increasing availability of sequence information, more and more molecular markers are becoming available for use in plant genotyping; for example SSRs (simple sequence repeats) and SNPs (single nucleotide polymorphisms) [1]. These markers are widely used in screening genebank collections of cultivated and wild germplasm, genome mapping for traits of interest and marker assisted selection for the development of new cultivars. Automatic fluorescent detection systems and related software for fragment size analysis and data tracking have made high throughput genotyping possible [2]. Management of large amounts of genotype data can be a difficult task without the support of information management systems, even for laboratories with only moderate throughput. A number of Laboratory Information Management Systems (LIMS) for sequencing data are available, while LIMS for genotyping data are more rare. However, many of these are expensive commercial products that provide solutions beyond what is often needed, use commercial databases and have several dependencies. Some freely available information management systems have been built for genotyping or for functional genomics, such as software to manage TaqMan SNP genotyping data [3], the GenoDB [4], PacLIMS [5] and SNPP [6] each with different levels of dependencies and functionalities. While many are specific to the genotyping of SNPs or pertaining to functional genomics, some like GenoDB are data management systems for microsatellite markers and linkage analysis with functionalities tuned to human genotyping projects and running on Windows based platforms. Based on the lack of a suitable system, we initiated a project to build a LIMS that could track the genotype workflow of projected moderately high throughput (10–50,000 samples/month) laboratories such as at ICRISAT and in many national programs, which was inexpensive, easy to use and would allow for future modifications.

Managing genotyping data can be a challenge, given the nature of the workflow. It is possible for samples to fail at different steps of the workflow, resulting in a need to repeat the processing of these samples. Where the allele calling/binning is not accurate, data may have to be replaced with data from a repeated assay. Records for a particular sample may need to be revised over time; thus the management system needs to keep track of the sample DNA from its source to the genotyping data/files. Moreover, entire steps in the workflow may be found redundant as researchers find alternatives to certain steps of the process. Thus the requirements of the LIMS system are many; it must be modular so that it is possible to skip certain modules in the workflow, or add new modules that would aid in the process of data management. Besides serving as a workflow manager, the system must also provide visible quality checks and centralization of data. The ability to track data and communicate quality information gives the laboratory the tools to improve methods and work practices.

We report here the development and implementation of the AGL-LIMS (Applied Genomics Laboratory-LIMS) that provides for quality control in a medium scale genotyping laboratory. The primary users of the application are laboratory technicians and students who have very limited amount of time to learn before they start entering their data. Thus the data entry and retrieval functions are simple with a built-in error checking facility. Access to the database is through the browser. The AGL-LIMS also implements functions for the automated binning of alleles, eliminating the errors introduced by incorrect allele sizing. The AGL-LIMS ensures that all samples have been treated identically, and all data entered or edited have date and time stamps. The final output files and allele binning output are available to the user for analysis. Since capillary electrophoresis may not always be the method of choice for all crops, the application also provides storage and retrieval functions of gel electrophoresis data. The entire application with the database and tutorial is available for download, installation and modification to suit other's needs.

## Implementation

The AGL-LIMS has been implemented with open source freely available software. The client accesses the system through the browser. The GUI and middleware have been implemented using Java Struts Framework technologies. Two versions of the software are available, one that uses the MS-SQL 2000 server for data storage and another using the PostgreSQL server. The application has also been tested with the free SQL Server 2005 Express Edition. The Tomcat apache web server is used to provide GUI interconnections with the database. The modular nature of development of the software ensures that as changes arise, they will be easier to isolate and carry out without having to rewrite entire code. The application is also platform independent. Secure user access is ensured through a web browser when the user logs into the system and their identity is associated with all subsequent actions. The system provides for hierarchical storage of data where one project may contain several studies and each study can comprise of several experiments. A Flash tutorial is available, written using the wink freeware [7].

## Modules

**Figure 1:**
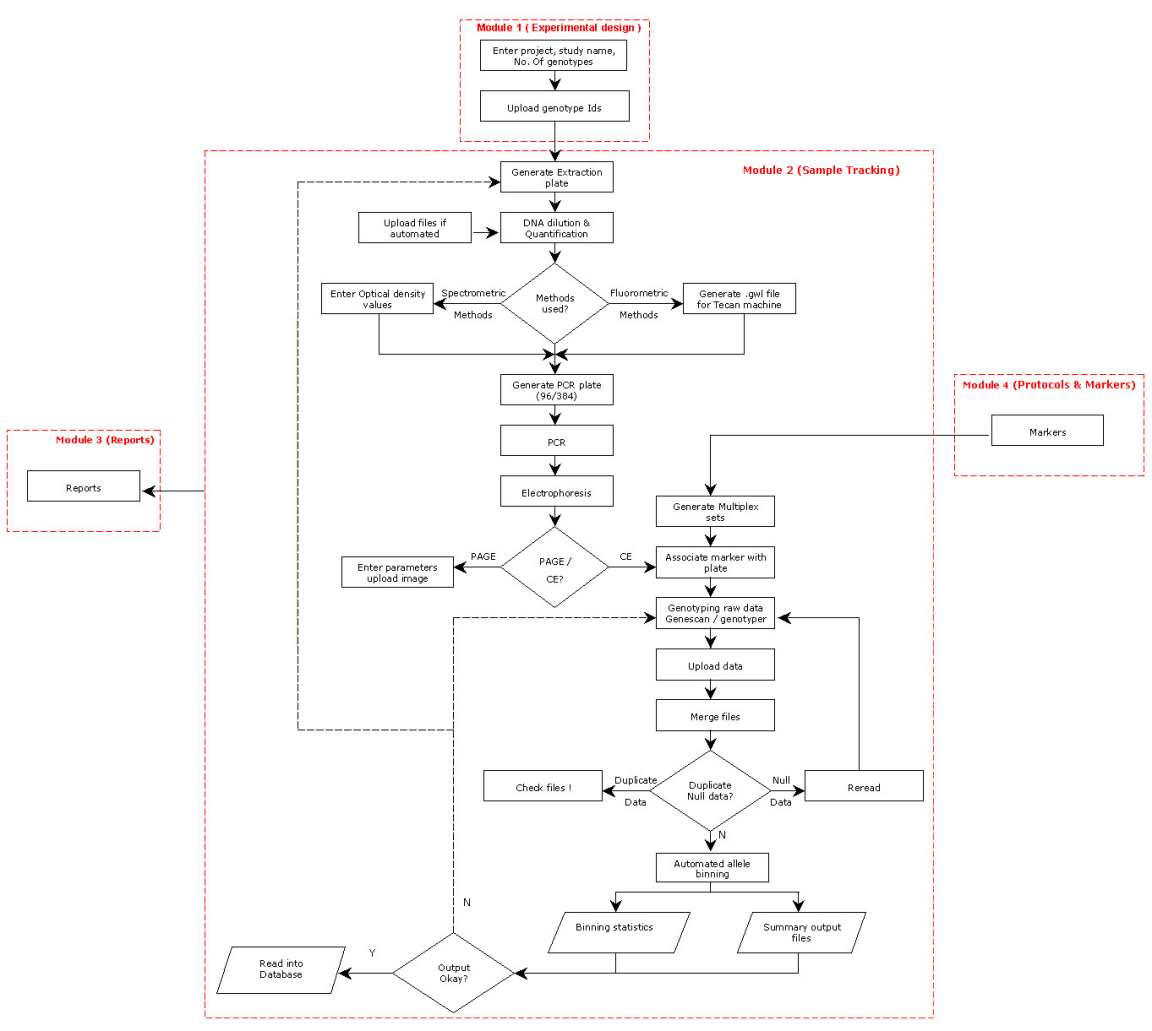
LIMS modules and information flow. Dotted rectangles indicate the four LIMS modules. Rectangles indicate processes in the data flow chart, diamond indicates decision, and parallelogram indicates data storage steps. (PCR: Polymerase Chain Reaction, PAGE: Polyacrylamide Gel Electrophoresis, CE: Capillary Electrophoresis)

The AGL-LIMS is composed of four main modules: (a) experimental design, including upload of populations used for the genetic characterization of germplasm (b) sample tracking of DNA extractions, DNA quantification and normalization, PCR marker characterization through gel electrophoresis as well as capillary electrophoresis, upload of genotype data and auto-binning of allele sizes (c) generation of reports and (d) storing of data on markers/protocols. Figure 1 describes the four major modules and the flow of information in the system. The roles of each of the LIMS modules are described below.

### Experimental design

The user enters a name for the project, study and the experiment along with the number of accessions being screened. Data on genotype identities/names must be uploaded at this stage, since the LIMS uses the genotype identifiers to 'create' a DNA extraction plate. Germplasm passport data or characterization data if available may also be uploaded into the system at this stage.

### Sample tracking

This module leads the user through the steps of genotyping workflow, from Sample DNA localization in microtitre plates, DNA quantification and normalization, PCR amplification using information held in the database for each marker. Markers are characterized through polyacrylamide gel electrophoresis (PAGE) as well as capillary electrophoresis (CE). A prior step to characterization for capillary electrophoresis is the assignment of multiple sets of markers based on allele size data or dye-colours. Finally users can upload stained PAGE images or capillary electrophoresis output files, output from downstream analysis with genotyper software, and merge files across experiments for automated allele size binning.

The LIMS supports management of some of the issues that arise during high-throughput genotyping, such as the treatment of samples that failed during electrophoresis. Genotyping results for these samples can be replaced with data from repeat assays. The system allows elimination of duplicate data by alerting the user to the existence of two records for the same genotype and seeks user intervention to decide which one to eliminate.

The relational database is comprised of 27 tables that are linked with each other through primary keys. The tables store data entered at each step of the sample tracking process besides genotyping output files, protocols and marker details. The LIMS Id uniquely identifies each record in the database and is used for trace back function. Each project may have several study names associated with it. Each study may have several experiments. Multiplex marker sets generated in a study can be reused for all experiments in that study. Once the user logs in, he may work through a study and all the experiments associated with that study during a single session.

#### Automated allele binning

**Figure 2:**
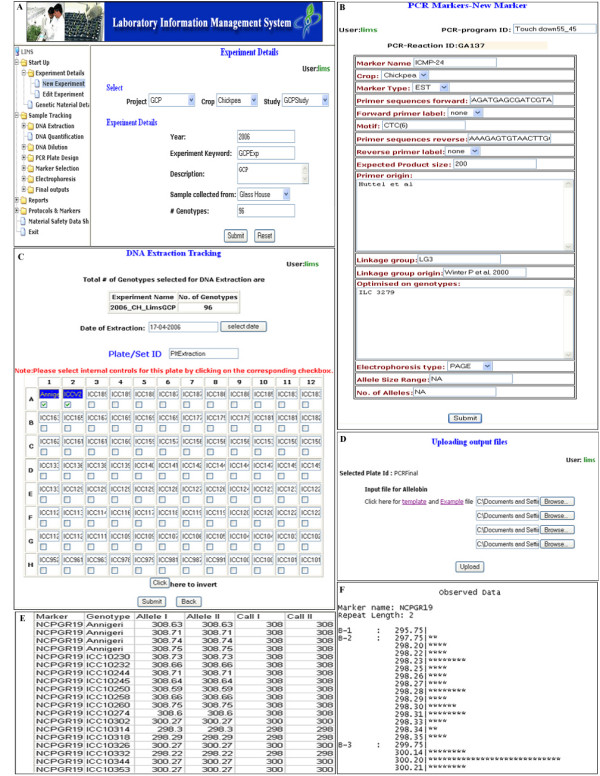
LIMS modules and user interfaces. **A**: The LIMS user interface for *Experimental Design*. **B**: *Protocols and Markers *– Form uploads for information on markers. **C**: *Reports *– sample DNA localization in microtitre plates for DNA extraction. **D**: *Sample Tracking *– User interfaces to upload capillary electrophoresis output files. **E & F**: Output files from the allele-binning program.

During SSR genotyping, inconsistencies in allele labels arise when software like the ABI-Prism Genotyper are used for allele calling. These software are mostly semi-automatic and require manual intervention. To make the allele labeling uniform, the data in terms of non-integer base pair values obtained from fragment analysis software by the user are loaded into the system as Excel sheets. These sheets can then be merged across experiments in a study. The user is alerted if duplicates or null calls are present, otherwise the merged files may be submitted to an allele-binning programme that automatically classifies allele sizes into discrete bins. The "Allelobin" programme incorporated within the LIMS is a variation of the method of Idury and Cardon [8], rewritten in Java. The variation introduced concerns the use of additional statistics like median and median absolute deviation (MAD) as a measure of dispersion and the additional rounding off of the bin median to preserve genetically expected repeat lengths of the marker. The user may download the output from this program, which includes a summary output file containing the newly called alleles, summary statistics and a histogram output (Figure 2, panels E and F). The output data is transferred to the database once flagged by the user.

### Generation of reports

Data submitted to the database can be viewed or printed as reports from this module. The reports may also be downloaded as tab delimited files. These include DNA and PCR plate design, DNA quantification results, and details of PCR markers, programs and reagent calculations.

### Data storage

Information on DNA extraction, quantification and PCR protocols entered through the "Protocols and Markers" module is archived here. Simple forms allow the user to enter details on protocols used as well as details about the markers type, primer sequences, reagent concentrations and temperature cycle profiles, product size and mapped assignments to linkage groups.

### Application use, optimization, future directions

The application came into routine use at ICRISAT's Applied Genomics Laboratory in 2005, for the capture of genotyping data from chickpea, groundnut, sorghum and pigeonpea and is being continually optimized to achieve all desired functionality without compromising on speed. Training of bench personnel is also being taken up to increase LIMS familiarity and usage. Future directions include automating the data entry process, allowing for bar code reading of 96 and 384 well plates for those laboratories where such facilities exist and incorporating a component for inventory management of reagents and other laboratory consumables.

## Conclusion

The application presented here is suitable for the management of medium to large quantities of genotyping data, such as that obtained with high throughput genotyping system using facilities such as Tecan^® ^Liquid-handling robotics with DNA plate reader (SPECTRAFluor Plus) for DNA quantification and normalization and ABI Prism 3700 DNA Analyser for genotyping. The functions of this application include generation of DNA and PCR plate designs, creation of files that can be read by the Tecan robotics and Applied-Biosystems software; checking for data duplicates, merging of files and auto binning of allele sizes, import of raw genotype data during the genotyping workflow etc. The application also allows replacement of records with repeat experiments and checks for null data and allows the user to trace back to the source of the raw data in the capillary electrophoresis files. The application has user-friendly interfaces making it easy to learn and use. While the application has been designed for the local user who carries out genotyping of microsatellite markers, the application being modular can be extended or modified to suit a laboratory's specific needs. The application is freely available to academic users.

## Availability and requirements

While the MS-SQL version of the software has been validated on the Windows platform, the postgreSQL version has been validated on Windows and Linux. Both the MS-SQL version as well as the postgreSQL version of the LIMS may be downloaded from the project home page:  along with the installation instructions. A sample dataset is available with the software package. The application and code is available to academic users without restriction. The application is also available for testing at  for a limited period. The user may log in with the password 'lims'.

## Authors' contributions

PTR, LY, SP and SL coded the software. HKB, DAH, EKE, EM, SK, SS, RF, RKV provided design, testing, and feedback. JHC and MV were involved in development of the concept, RR, PVP and SC for rewriting the allele binning algorithm; and JB in the design of GUI and quality parameters, JB drafted and edited the manuscript. All authors read and approved the final manuscript.

## References

[B1] Rudd S, Schoof H, Mayer K (2005). PlantMarkers – a database of predicted molecular markers from plants. Nucleic Acids Res.

[B2] Cryer NC, Butler DR, Wilkinson MJ (2005). High throughput, high resolution selection of polymorphic microsatellite loci for multiplex analysis. Plant Methods.

[B3] Monnier S, Cox DG, Albion T, Canzian F (2005). T.I.M.S: Taqman Information Management System, tools to organize data flow in a genotyping laboratory. BMC Bioinformatics.

[B4] Li J-L, Deng H, Dong-Bing L, Fuhua X, Chen J, Gao G, Recker R, Deng H-W (2001). Toward high-throughput genotyping: dynamic and automatic software for manipulating large-scale genotype data using fluorescently labeled dinucleotide markers. Genome Res.

[B5] Donofrio NM, Rajagopalan R, Brown DE, Diener SE, Windham DE, Nolin S, Floyd A, Mitchell TK, Galadima N, Tucker S, Orbach MJ, Patel G, Farman ML, Pampanwar V, Soderlund C, Lee Y-H, Deen RA (2005). PACLIMS: A component LIM system for high throughput functional genomic analysis. BMC Bioinformatics.

[B6] Zhao L-J, Li M-X, Guo Y-F, Xu F-H, Li J-L, Deng H-W (2005). SNPP: automating large-scale SNP genotype data management. Bioinformatics.

[B7] Debugmode Wink. http://www.debugmode.com/wink/download.php.

[B8] Idury RM, Cardon LR (1997). A simple method for automated allele binning in microsatellite markers. Genome Res.

